# Swinepox Virus Strains Isolated from Domestic Pigs and Wild Boar in Germany Display Altered Coding Capacity in the Terminal Genome Region Encoding for Species-Specific Genes

**DOI:** 10.3390/v13102038

**Published:** 2021-10-09

**Authors:** Franziska K. Kaiser, Anastasia Wiedemann, Bianca Kühl, Laura Menke, Andreas Beineke, Wolfgang Baumgärtner, Peter Wohlsein, Kerstin Rigbers, Paul Becher, Martin Peters, Albert D. M. E. Osterhaus, Martin Ludlow

**Affiliations:** 1 Research Center for Emerging Infections and Zoonoses, University of Veterinary Medicine Hannover, 30559 Hannover, Germany; franziska.kaiser@tiho-hannover.de (F.K.K.); Laura.Menke@helmholtz-hzi.de (L.M.); albert.osterhaus@tiho-hannover.de (A.D.M.E.O.); 2 Institute for Virology, University of Veterinary Medicine Hannover, 30559 Hannover, Germany; anastasia.wiedemann@tiho-hannover.de (A.W.); paul.becher@tiho-hannover.de (P.B.); 3 Department of Pathology, University of Veterinary Medicine Hannover, 30559 Hannover, Germany; bianca.kuehl@tiho-hannover.de (B.K.); andreas.beineke@tiho-hannover.de (A.B.); Wolfgang.Baumgaertner@tiho-hannover.de (W.B.); Peter.Wohlsein@tiho-hannover.de (P.W.); 4 Chemisches und Veterinäruntersuchungsamt Karlsruhe, 76187 Karlsruhe, Germany; kerstin.rigbers@cvuaka.bwl.de; 5 Chemisches und Veterinäruntersuchungsamt Westfalen, 59821 Arnsberg, Germany; Martin.Peters@cvua-westfalen.de

**Keywords:** swinepox virus, domestic pig, wild boar, next generation sequencing, persistence, intrauterine infection

## Abstract

Swinepox virus (SWPV) is a globally distributed swine pathogen that causes sporadic cases of an acute poxvirus infection in domesticated pigs, characterized by the development of a pathognomonic proliferative dermatitis and secondary ulcerations. More severe disease with higher levels of morbidity and mortality is observed in congenitally SWPV-infected neonatal piglets. In this study, we investigated the evolutionary origins of SWPV strains isolated from domestic pigs and wild boar. Analysis of whole genome sequences of SWPV showed that at least two different virus strains are currently circulating in Germany. These were more closely related to a previously characterized North American SWPV strain than to a more recent Indian SWPV strain and showed a variation in the SWPV-specific genome region. A single nucleotide deletion in the wild boar (wb) SWPV strain leads to the fusion of the SPV019 and SPV020 open reading frames (ORFs) and encodes a new hypothetical 113 aa protein (SPVwb020-019). In addition, the domestic pig (dp) SWPV genome contained a novel ORF downstream of SPVdp020, which encodes a new hypothetical 71aa protein (SPVdp020a). In summary, we show that SWPV strains with altered coding capacity in the SWPV specific genome region are circulating in domestic pig and wild boar populations in Germany.

## 1. Introduction

Swinepox virus (SWPV) is the only member of the genus Suipoxvirus, which belongs to the subfamily Chordopoxvirinae, within the family Poxviridae. This virus contains a linear double-stranded DNA genome of 146 kbp and is the etiological agent of an eruptive dermatitis in pigs, known as swinepox. Swinepox was first described as a disease of domestic pigs in Europe in 1842 [1] and in the USA in 1929 [2] but is now known to have a worldwide distribution and is endemic in many areas of Africa [3], Australia [4], North America [5], South America [6,7] and Asia [8,9]. For a number of decades, Vaccinia virus (VACV) was the etiological agent of a similar disease in domestic pigs with distinctive pustular lesions [10]. However, the incubation period of VACV infection was shorter than that of SWPV infection with lesions that were in general smaller in size [11,12]. Although VACV- related viruses continue to circulate in some countries such as Brazil and cause occasional disease in wildlife and domestic animals [13,14,15], VACV is not endemic in Western Europe and can be readily excluded as a causative agent by SWPV-specific molecular diagnostic assays [14,16].

Clinical studies, experimental infection of laboratory animals and in vitro infection of cell lines originating from different species have shown that SWPV displays a high degree of host specificity with infections restricted to domestic pigs [17] with a single case reported in a wild boar [18]. Experimental infection of other species with SWPV including rats, mice and rabbits have been unsuccessful with respect to induction of viremia or skin lesions pathognomonic of swinepox [19]. Similarly, infection of non-porcine mammalian or avian cell lines failed to produce viral particles [20]. Piglets under three months of age are most commonly infected and display more severe clinical signs of infection [17,21]. The macroscopic manifestations of swinepox have been described as a multifocal, eruptive dermatitis with cutaneous lesions commonly observed on the abdomen, inner surface of the legs, pinnae and sporadically on the face [3,4,22]. Clinical lesions are restricted to the skin and less frequently, mild changes in the superficial lymph nodes have been reported [10,23]. Histological examination of tissues from swinepox-infected pigs has shown that virus replication occurs in keratinocytes of the epidermal stratum spinosum [5,12,24]. The combination of a strongly restricted host range and genetic stability aroused interest in SWPV as a vaccine expression vector for the immunization of swine [25,26,27].

In addition to direct contact between infected and susceptible animals, a mechanical route of transmission by insect vectors such as the swine louse (*Haematopinus suis*) has been shown to facilitate the spread of the virus between populations [28]. Early experiments investigated the role of *Haematopinus suis* in swinepox infections and demonstrated its function as a mechanical vector but not as an intermediate host [29]. Additionally, the clinical appearance of morphological and histological signs of a congenital SWPV infection and thereby the possibility of a vertical transmission of SWPV in naturally occurring infections has been described [30,31]. Morbidity of SWPV infection can be high in piglets of an infected litter, but mortality is often low. This has resulted in a paucity of research into this virus since it is primarily connected to poor sanitation and plays no major economical role in modern agriculture of developed countries [32].

SWPV shares genomic similarities with virus species from other poxviral genera such as hairpin-shaped inverted terminal repetitions [33] and genes that modulate antiviral immune responses [34]. However, little is known about the strain diversity of SWPV due to the limited numbers of complete genome sequences of SWPV. Three open reading frames (ORFs) are unique to the SWPV genome (SPV018, SPV019, SPV020) and encode proteins containing 63–73 amino acids which have been postulated to play a key role in the pathogenesis of swinepox [35]. In this study, we have characterized SWPV strains isolated from German domestic pigs and wild boar to investigate genome sequence diversity and draw conclusions on horizontal and vertical transmission routes and possible reservoirs.

## 2. Materials and Methods

### 2.1. Patholomorphological, Histological and Ultrastructural Examination

Tissue samples from domestic pigs (*Sus scrofa domesticus*) were collected from recurrent sporadic cases of swinepox that occurred in piglets on two conventional pig farms in Westphalia, Germany between July 2019 and January 2020. A complete postmortem examination was performed on three piglets with suspected poxvirus skin lesions and tissue samples (skin, tongue, esophagus, lung, heart, liver and intestine) were fixed in 4% buffered formaldehyde and routinely processed for histopathological examination by embedding in paraffin wax according to standard procedures, prior to haematoxylin–eosin (HE) staining of microtome-cut tissue sections. A formalin fixed block containing skin tissue from a previously described swinepox case in a domestic pig in 2008 was also used for retrospective SWPV genome analysis [36]. Native tissue samples were also harvested and preserved frozen for virus culture and molecular biological investigation. Additional frozen skin samples were obtained from two SWPV-infected juvenile wild boar (*Sus scrofa*) found in Baden-Württemberg with an eruptive dermatitis in October 2019. Ultrastructural examination was performed using a standardized diagnostic electron microscopy protocol with negative staining technique according to the recommendations of the Robert Koch Institute, Germany. Briefly, the unfixed skin lesions were scarified and placed against formvar-filmed electron microscopy copper grids to adsorb virus particles. These were negatively contrasted with 3% (*w*/*v*) phosphotungstic acid for 30 s and then ultrastructural investigation performed using an LEO0906 Electron microscope (Carl Zeiss, Oberkochen, Germany). Samples were initially examined using a 50,000× magnification.

### 2.2. Cells and Viruses

Porcine embryonic kidney cell line SPEV (cell line 0008, Friedrich Loeffler Institute, Germany), porcine lymphoma cell line 38A_1_D [37], porcine kidney cell lines SK6 [38], PK-15 cells and PK-15 derivative Riebe 5-1 (Friedrich Loeffler Institute, Germany) and a porcine testis epithelia cell line Riebe 255 (Friedrich Loeffler Institute, Germany) were maintained in Eagle’s minimal essential medium (EMEM) (Thermo Fisher Scientific, Waltham, MA, USA) containing 10% fetal bovine serum (FBS) (Thermo Fisher Scientific) except for PK-15 cells in which 7.5% FBS was used. PK-15 cell lines were confirmed to be free of porcine circovirus type 1 and 2 contamination using specific primer pairs in a PCR performed on DNA extracted from cells [39,40]. Virus isolation was performed using skin samples containing lesions from a domestic piglet (Host ID 201-20046) or wild boar piglet (Host ID 201-20070) with suspected SWPV infection. Tissue samples were homogenized with PBS and centrifuged at 12,000× *g* for 5 min at 4 °C. The clarified supernatant was used to inoculate PK-15 cells for 90 min at 37 °C. Following infection, the medium was changed to EMEM containing 10% FBS and 1% Penicillin, Streptomycin and amphotericin and infected cell monolayers were passaged every three days until cytopathic effects (CPE) were observed.

### 2.3. Detection of SWPV-Specific Sequences Using PCR and qPCR

A SWPV-specific real-time quantitative PCR (qPCR) was performed to confirm SWPV infection in clinical samples using probe and primer pair sequences targeting the C20L-C1L region [41]. The oligonucleotide sequences used were 5′-TAATCCGGGCATCAATCCTC-3′ (forward primer), 5′ GCTGATTGGGCCAGAAAATG-3′ (reverse primer) and 5′ FAM TTCCCTCCACAGCTGCAAATGCTACT-TAMRA-3′ (probe). All available samples from SWPV-infected animals or cell cultures were homogenized, centrifuged, and clarified supernatants were collected. DNA was extracted from frozen tissue and a single formalin fixed tissue block using a QIAmp DNA Mini kit and QIAamp DNA FFPE Tissue Kit (Qiagen, Hilden, Germany), respectively, according to the manufacturer’s instructions. Amplification of SWPV-specific sequences via qPCR was performed using 45 cycles and an annealing temperature of 53 °C following the recommended protocol of the Luna Probe One-Step qPCR kit (NEB, Ipswich, MA, USA). A specific 238 bp region (12,989–13,227 bp) of the SPV020 ORF was amplified by PCR using the primer pair 5′ GAAGATATTGACACTGTATCCATAC-3′ and 5′ GAGCACTACATTTCATTTC-3′ using the Q5^®^ High-Fidelity PCR kit (NEB). For Sanger sequencing of ORF SPV006 and SPV136 coding sequences, two primer pairs were used, 5′-TGAACGGAATCTGAAATACGA-3′ and 5′-AAATATCTCATACAATCATTATACTTAC-3′ for SPV006 and 5′-ATTACAGGAAAGATTGGCGTA-3′ and 5′-TAATTTCCAAGACCTTCGCTT-3′ for SPV136 [9].

### 2.4. Permissivity of Different Porcine Cell Lines to SWPV Infection

The isolated virus strains from domestic pigs and wild boar were used to test the permissivity of porcine-derived cell lines. Monolayers of the kidney-originated cell lines (SPEV, SK6, and two PK-15 subclones) as well as of the 38A_1_D lymphoma cell line and porcine epithelia testis cell line Riebe-255 were infected with the two SWPV strains for 2 h at 37 °C. The cell cultures were maintained in EMEM supplemented with 10% FBS and 1% penicillin/streptomycin and monitored for the development of cytopathic effects. Following the infection, supernatant samples were taken at days 0, 3, 6 and 10 post-infection and the amount of viral DNA was quantified by SWPV-specific qPCR with three replicates.

### 2.5. Whole Genome Sequencing and Analysis

Next generation sequencing (NGS) was used to obtain the complete genome of the SWPV strains isolated from the domestic pig (Host ID 201-20046; Table 1) and wild boar (Host ID 201-20070; Table 1). DNA Library preparation was performed with an Illumina Nextera TruSeq Library Preparation Kit (Illumina, Inc, San Diego, CA, USA) followed by sequencing on a NextSeq 550 sequencer to obtain 2× 10Mio reads (150 bp, paired end) per sample. Full-genome sequences were compiled and analyzed using QIAGEN CLC Genomics Workbench (v12). Annotation of ORFs was performed using Geneious Prime (Biomatters, Ltd., Auckland, New Zealand).

### 2.6. Phylogenetic Analysis

The phylogenetic relationship of different SWPV strains was investigated by performing a multiple sequence alignment using MAFFT version 7 [42] of the two newly generated full genome sequences (SWPV/domestic/GER/2019; GenBank accession no. MZ773481 and SWPV/wildboar/GER/2019, GenBank accession no. MZ773480) and the two existing SWPV genomes deposited in GenBank (GenBank accession no. NC_003389.1 and MW036632). The maximum likelihood method was applied using MEGA X [43] with 1000 bootstraps and the Tamura–Nei model [44] was used to perform the phylogenetic analysis based on the whole genome sequences. Branch lengths drawn to scale on the phylogenetic tree represent the number of substitutions per site. The phylogenetic trees for the evolutionary investigation of the nucleotide similarity of the open reading frames SPV006 (Hasegawa–Kishino–Yano model [45]) and SPV136 (Tamura–Nei model) were generated in an analogous manner.

## 3. Results

### 3.1. Clinical and Pathological Findings

**Figure 1:**
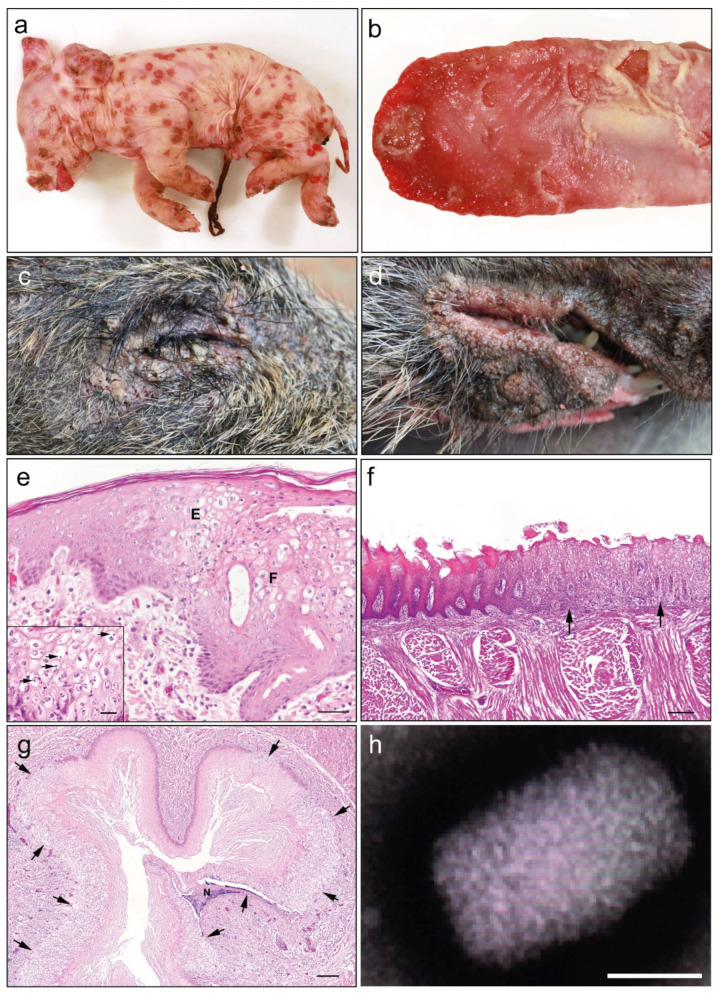
Pathological and ultrastructural investigation of tissues from animals diagnosed with SWPV infection. (**a**) Macroscopic skin lesions visible as multifocal severe, erythematous maculae on a SWPV-infected piglet. (**b**) Tongue of a one-day-old SWPV-infected piglet with multifocal coalescing vesicular to bullous lesions and consecutive erosions. (**c**,**d**) Macroscopic skin lesions consisting of multiple crusted pustules surrounding the eye (**c**) and lip (**d**) of a juvenile wild boar. (**e**) Skin of a one-day-old piglet infected with SWPV characterized by ballooning degeneration of the epidermal (E) and follicular (F) epithelial cells; HE, bar = 100 µm; inset: higher magnification reveals cytoplasmic eosinophilic inclusion bodies in degenerated epidermal cells (arrows); HE, bar = 20 µm. (**f**) Tongue of a one-day-old piglet infected with SWPV characterized by focal severe ballooning degeneration of the epithelial cells (arrows); adjacent regular epithelial lining; HE, bar = 200 µm. (**g**) Esophagus of a one-day-old piglet infected with SWPV characterized by multifocal severe ballooning degeneration of the epithelial cells (arrows); focal intraepithelial necrosis (N); HE, bar = 200 µm. (**h**) Ultrastructure of a characteristic orthopoxvirus particle in the skin of a piglet demonstrated by negative staining, bar = 100 nm.

Three piglets with skin lesions pathognomonic for poxvirus infection originating from two farms in North Rhine Westphalia, Germany in July 2019 and January 2020 were submitted to the local veterinary state laboratory (CVUA WFL, Arnsberg) for further investigations into the etiology of the disease. Two piglets from farm 1 (201-200046, 201-200047) died within 24 h of birth while one piglet from farm 2 (201-200045) was euthanized on day 1 post-birth (Table 1). Approximately ten litters of domestic pigs had been affected with this disease syndrome on farm 1 in the previous 18 months and involved one or two symptomatic piglets per litter, which died within 24 h of birth. On farm 2 only two litters with a total of three symptomatic piglets had been noted in the previous 18 months. On both farms, the sows had intermittent contact with one another, and animals were kept in year-round stable housing and routinely treated against ecto- and endoparasites using defined regimes. Post-mortem examination of all domestic piglets showed concordantly multifocal severe, erythematous maculae covering the whole body (Figure 1a). These lesions were characterized by round, occasionally coalescing papules with raised, wall-like borders, encircling a depressed center, and partially covered by encrusted exudate. Circumscribed epithelial defects were observed additionally on the tongue (Figure 1b). Additional tissue samples were obtained from two juvenile wild boar with suspected poxvirus lesions submitted to the local veterinary state laboratory (CVUA Karlsruhe) in Baden-Württemberg in October 2019. Post-mortem examination revealed extensive skin lesions consisting of papules around the eyes and lips of the wild boar (Figure 1c,d). Microscopic examination of tissue sections from the domestic piglets showed a multifocal, moderate to severe, proliferative, and necrotizing dermatitis and folliculitis with ballooning degeneration and eosinophilic cytoplasmic viral inclusions were found (Figure 1e). A moderate perivascular and periadnexal lymphohistiocytic and plasmacytic infiltration was present in the dermis. The tongue showed a multifocal ulcerative glossitis with adjacent epithelial hyperplasia, ballooning degeneration and cytoplasmic viral inclusions (Figure 1f). Esophageal lesions consisted of focal epithelial proliferations with ballooning degeneration and cytoplasmic viral inclusions (Figure 1g). Other organs and tissues were without morphological changes. Electron microscopical investigation showed evidence of 220 × 450 nm sized, brick-shaped orthopoxvirus particles, with an electron-dense DNA core containing a biconcave core in tissue from skin lesions of all necropsied piglets (Figure 1h). The tissue tropism was further investigated by performing qPCR using SWPV-specific primers on DNA extracted from different organs of a congenitally infected piglet (Host-ID 201-20047). The lowest ct values were found in skin, umbilical cord tissue and tongue with higher ct values noted in the lung and intestine (Table 1). SWPV was not detected by qPCR in tissue samples from liver or kidney. SWPV was also detected in frozen skin samples from two additional recent cases of congenitally infected piglets (Host-IDs 201-200045 and 201-200046) and an FFPE skin sample from a 2008 case of a SWPV-infected piglet (Host-ID 101-200136). Skin samples collected in 2019 from two juvenile wild boar (Host-IDs 201-200070 and 201-200071) with characteristic swinepox lesions were also positive by qPCR for SWPV with low ct values of 14.16 and 15.95 respectively.

### 3.2. Assessment of The Susceptibility of Porcine Cell Lines to SWPV Infection

**Figure 2:**
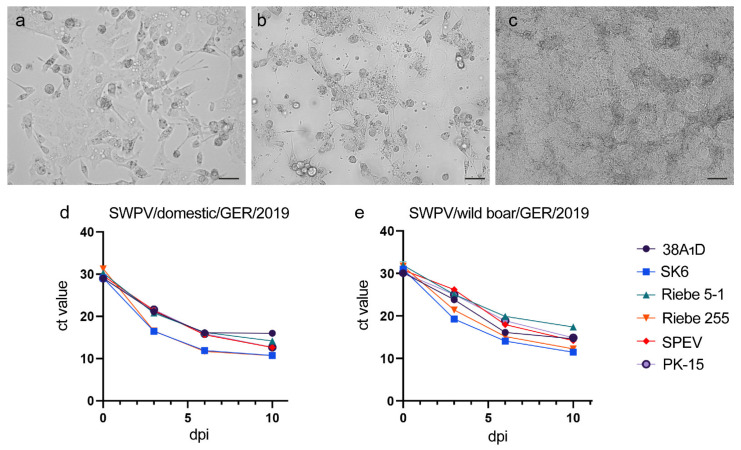
SWPV infection of porcine cell lines. (**a**–**c**) Characteristic cytopathic effects including cell rounding, ballooning and vacuolization were observed in PK-15 cells infected with a German domestic pig SWPV strain (SWPV/domestic/GER/2019) (**a**) and German wild boar SWPV strain (SWPV/wildboar/GER/2019) (**b**), but not in mock-infected PK-15 cells (**c**). Scale bars, (**a**,**b**) 30 µm, (**c**) 75 µm. (**d**,**e**) Porcine kidney cell lines (SPEV, SK6 and two PK-15 subclones), porcine testis epithelia cells (Riebe-255) and a porcine lymphoma cell line (38A_1_D) were infected with SWPV/domestic/GER/2019 (**d**) and SWPV/wildboar/GER/2019 (**e**). Supernatants were collected at three, six and ten days post-infection and ct values were determined in triplicate by qPCR.

Virus isolation from confirmed swinepox cases was performed by infection of PK-15 cells with clarified supernatant from pox skin lesions that had been homogenized in PBS from a SWPV-infected domestic pig (Host-ID 201-20046) and wild boar (Host-ID 201-20070). Initially, no cytopathic effects were observed. Therefore, infected cells were blind-passaged until the characteristic CPE of SWPV including cell rounding, ballooning and vacuolization developed at passage four and three for the domestic pig (SWPV/domestic/GER/2019) and wild boar (SWPV/domestic/GER/2019) strains, respectively (Figure 2a–c). The susceptibility of additional porcine kidney cell lines (SPEV, SK6 and two PK-15 subclones), a porcine testis epithelia cell line (Riebe-255) and a porcine lymphoma cell line (38A_1_D) to infection with the German domestic pig and wild boar SWPV strains was investigated together with virus growth kinetics over time. In the absence of a SWPV-specific antibody and with cytopathic effects often absent or subtle in infected cell cultures, a SWPV-specific qPCR was used to assess virus replication. All tested cell lines showed evidence of SWPV replication as evidenced by a decreasing ct value (Figure 2d,e) with characteristic SWPV CPE observed in all cell lines by 10 dpi. The lowest ct values in the cell culture supernatant was attained ten days post-infections in the porcine kidney cell line SK6 (domestic pig SWPV strain: 10.7 ± 0.06; wild boar SWPV strain: 11.5 ± 0.06) followed by the porcine testis epithelia cell line Riebe 255 (domestic pig variant: 10.7 ± 0.08; wild boar variant: 12.3 ± 0.09).

### 3.3. Phylogenetic and Whole Genome Analysis of SWPV Sequences

**Figure 3:**
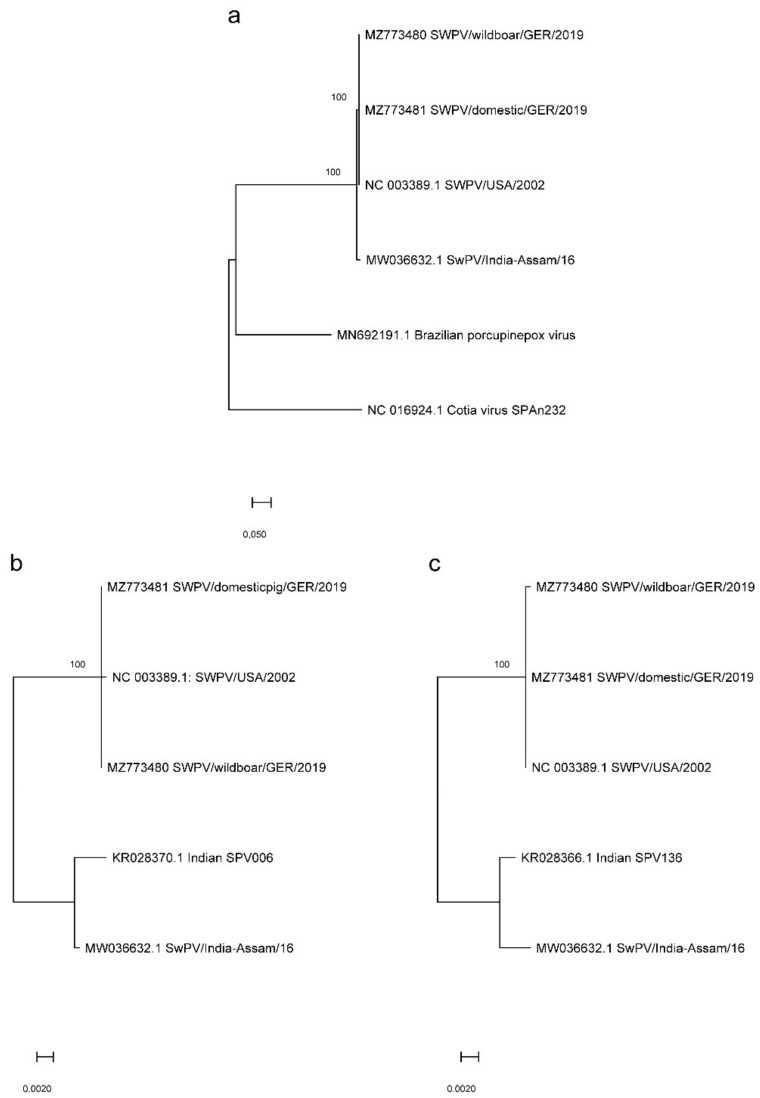
Phylogenetic analysis of German SWPV strains. (**a**) Maximum likelihood tree based on the full-length genome sequence of available SWPV sequences (SWPV/USA/2002, GenBank accession no. NC_003389.1 and SWPV/India-Assam/16, MW036632.1) and two full genome SWPV sequences generated in this study. Brazilian porcupinepox virus 1 strain UFU/USP001 (MN692191.1) and Cotia virus SPAn232 (NC_016924.1) were included as outgroups. (**b**,**c**) Maximum likelihood trees based on the nucleotide sequences of the SPV006 (**b**) and SPV136 (**c**) ORFs.

SWPV is the single member of the genus Suipoxvirus, within the subfamily Chordopoxvirinae and its clade is between that of members of the genus Capripoxvirus such as lumpy skin disease virus and the novel Brazilian porcupinepox virus. However, knowledge about the phylogenetic relationships and sequence diversity within the SWPV species is limited. We therefore compared the two available whole-genome SWPV sequences from domestic pigs in North America (SWPV/USA/2002, GenBank accession no. NC_003389.1) and India (SWPV/India-Assam/16, GenBank accession no. MW036632.1) to the new German genome SWPV sequences obtained in this study. Phylogenetic analysis showed that the German domestic pig and wild boar SWPV strains from 2019 form a clade with SWPV/USA/2002 and branch separately from SWPV/India-Assam/16 (Figure 3a). The SWPV strains derived from a German domestic pig and wild boar were found to have 99.924% identity with respect to nucleotide sequence (Table S1). These strains also showed 99.92% (wild boar)/99.94% (domestic pig) nucleotide sequence identity to SWPV/USA/2002 and 98.14% (wild boar)/98.15% (domestic pig) to SWPV/India-Assam/16 (Figure S1). Further phylogenetic analysis based on the nucleotide sequences of the SPV006 and SPV136 ORFs confirmed the existence of two separate clades of SWPV (Figure 3b,c).

**Figure 4:**
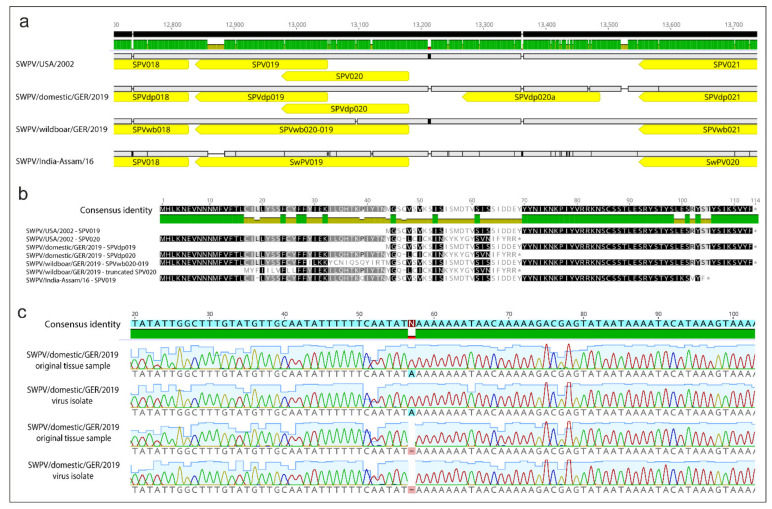
Analysis of SWPV specific genes in domestic pig and wild boar strains. (**a**) Schematic representation of the SPV019 and SPV020 ORFs present in different SWPV strains. Fusion of these genes via different single nucleotide deletions is apparent in the Indian domestic pig strain (SWPV/India-Assam/16, GenBank accession no. MW036632) and German wild boar SWPV strain (SWPV/wildboar/GER/2019, GenBank accession no. MZ773480). The SPV019 and SPV020 ORFs are present as separate overlapping ORFs in the American domestic pig strain (SWPV/USA/2002, GenBank accession no. NC_003389) and German domestic pig strain (SWPV/domestic/GER/2019, GenBank accession no. MZ773481). (**b**) Amino acid alignment between the SPV019 and SPV020 ORFs of different SWPV strains. A truncated variant of SPV020 is also present in the German wild boar SWPV genome and a novel ORF (SPVdp020a) exists downstream of SPV020 in the German domestic pig SWPV genome. (**c**) Analysis of DNA extracted from tissues samples from swinepox cases shows evidence of a single nucleotide deletion at position 13,096 bp in SPV020 ORF in the genome of a German wild boar SWPV strain from 2019 in both the virus isolate and the original tissue sample. This nucleotide deletion is not present in the isolate of the German domestic pig SWPV strain or in the original tissue sample. Alignments and analysis were performed using Geneious Prime (Biomatters, Ltd., Auckland, New Zealand).

**Figure 5:**
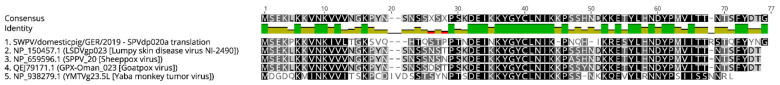
Alignment of the predicted amino acid sequence of a hypothetical protein encoded by the novel SPVdp020a present downstream of SPVdp020 in SWPV/domestic/GER/2019 with predicted proteins encoded by other poxviruses which display the closest predicted homology. The alignment and analysis were performed using Geneious Prime (Biomatters, Ltd.).

Comparison of whole-genome sequences of the two new German SWPV isolates showed 75 synonymous and 37 nonsynonymous changes, which were spread throughout the genome (Table S1). All previously annotated ORFs were present in these strains apart from some alterations in the SWPV-specific genome region. The wild boar SWPV strain but not the domestic pig SWPV strain was observed to contain a single base pair deletion at position 13,061 bp. This resulted in a frameshift in the SPVwb020 ORF, which led to the fusion of the SPVwb020 and SPVwb019 ORFs and the corresponding amino acid sequences, resulting in a hypothetical 113 aa SPVwb020-019 protein (Figure 4a). This represents the first 28 aa of SPVwb020, 15 unique aa and then 70 aa encoded by the SPVwb019 ORF. We confirmed that the single nucleotide deletion in the German wild boar SWPV strain did not occur because of in vitro passage by performing Sanger sequencing on the original tissue samples. A 238 bp region of SPVwb020-019 was amplified from DNA extracted from frozen tissue originating from the two SWPV cases in wild boar from 2019, the domestic pig cases from 2019 and a formalin-fixed tissue block from a SWPV case in a domestic pig from 2008. Subsequent analysis of Sanger sequencing data showed that the single base pair deletion in SPV020 was only present in tissue samples from the SWPV-infected wild boar piglets (Figure 4c). The single base pair deletion in the German wild boar SWPV strain also generates a truncated form of SPV020 (Figure 4b). This ORF encodes 51 aa, which represents 10 unique aa at the N-terminus with the remaining 41 aa identical to the corresponding region of SPV020 in other SWPV strains. The genome region encoding the three SWPV-specific genes (SPV018, SPV019 and SPV020) is followed by a 366 nt noncoding sequence. Within this sequence, a new ORF (SPV020a) encoding a hypothetical 71 aa protein was detected in the domestic pig SWPV sequence (Figure 4a). Further analysis showed that this ORF has homologs in other members of the Orthopoxviridae including a 58.9% similarity with the hypothetical protein LSDVgp023 from the lumpy skin disease virus (GenBank accession no. NP_150457.1) and with the hypothetical protein SPPV_20 from the sheeppox virus (GenBank accession no. NP_659596.1) (Figure 5).

## 4. Discussion

Outbreaks of swinepox are commonly observed in domestic pigs worldwide, but knowledge about the prevalence, strain diversity, wildlife reservoirs and evolutionary origins of SWPV is limited. In this study, SWPV strains isolated from skin samples containing pox lesions obtained from a wild boar piglet and a congenitally infected domestic piglet were sequenced and analyzed to determine the evolutionary origins of these strains. We observed a sequence divergence of only 0.076% (112 nt) between the SWPV strains present in domestic pigs and wild boar, meaning that at least two closely related strains are circulating in German wildlife and livestock pigs. In countries with a comparable, industrial structure of pig farming, the implementation of strict sanitary measures and high hygienic standards serves to shield the livestock population against contact with pathogens present in the environment and especially prevent potential direct or indirect encounters with wild boar. However, given the high nucleotide identity between the two SWPV strains, wild boar may have a role as a potential SWPV wildlife reservoir mediating the spread of SWPV to domestic pig populations. Additional sequencing of SWPV strains in domestic pig and wild boar populations is required to determine the routes of virus transmission.

The higher nucleotide sequence identity of the two German SWPV strains (2019) to the reference North American SWPV strain (2002) than to a recent Indian SWPV strain (2016) may reflect the history of domestication and distribution of the domestic pig. Zooarchaeological evidence shows that pigs were domesticated independently in at least two locations, namely Eastern Anatolia [46], from where the European domestic pig population originated [47] and China, from which it is assumed that domestic pig populations were then dispersed throughout Asia [48,49]. In North America, domestic pigs were introduced from Europe starting in the 15th century [50]. The two currently recognized SWPV lineages in Asia and Europe/North America might thus have evolved from SWPV strains present in wild pig populations following the two geographically separated domestication events. However, additional SWPV sequences from domestic pigs in more dispersed geographical regions worldwide would be required to confirm this hypothesis. It would also be of interest to obtain more full-length SWPV genome sequences from other wild pig species of the family Suidae present in Africa and Asia.

Genome analysis of the domestic pig and wild boar SWPV strains showed interesting differences with respect to potential alterations in gene expression in the region of the genome encoding SPV018, SPV019 and SPV020. These genes are unique to SWPV with no homologs within the wider Poxviridae family and are predicted to encode proteins that may be associated with immune evasion, restricted host range and/or virulence. The SWPV strain from wild boar had a one nucleotide deletion at position 13,061 bp resulting in fusion of the SPV019 and SPV020 ORFs. A similar fusion of the SPV019 and SPV020 ORFs has been recently observed in an Indian SWPV sequence from a domestic pig in which a single base pair deletion at position 12,993 bp caused a similar frameshift in SPV020 [51]. This sequence variation is thus not correlated with infection of domestic pigs or wild boar and additional genome sequences are required to determine the prevalence of fused SPV019 and SPV020 ORFs in SWPV strains originating from these species. A hypothetical protein (SPVdp020a) encoded by a novel ORF downstream of SPVdp020 in the German domestic pig SWPV strain has not been observed in other SWPV strains. However, further analysis showed that SPVdp020a displays approximately 58% homology to hypothetical proteins present in other related poxviruses including lumpy skin disease virus. Additional research is required to determine the function(s) of this and other unique SWPV proteins and the significance of mutations in this region of the genome for virus host range and pathogenicity. Interestingly, the SWPV-specific genome terminal region appears to be flexible with respect to gene expression in these hitherto unknown SWPV strains. These findings are compatible with sequencing studies on other poxviruses, demonstrating that terminal genome regions show significant variability and a disrupted collinearity in comparison to the conserved core region with approximately 80 genes [35,52].

The piggeries affected by this outbreak were assessed to be in a good hygienic condition with an absence of pig lice (*Haematopinus suis*) from the housing units, which indicates that vector-borne mechanical transmission was probably not responsible for the introduction of SWPV into these farms or subsequent virus transmission between animals. The pig louse has previously been shown to be a vector for SWPV transmission as the main lesions observed on infected pigs are on the abdomen and inner surface of the legs, corresponding to the predilection sites of these parasites. However, piglets which have been intravenously infected with SWPV also show lesions in the same positions [5]. The absence of this insect species in the housing units of the current swinepox cases suggests that the route of transmission was by subclinically or inapparently infected sows. This theory is supported by our findings of the permissivity of porcine lymphoma and epithelia cell lines and the detection of viral genomic sequence in different peripheral organs of the congenitally infected piglets. The establishment of a persistent poxviral infection has previously been reported for different orthopoxviruses [53,54,55], especially during ectromelia virus infections in mice, where bone marrow and blood cells appear to be key sites for persistence in immunocompetent mice [56]. Poxviruses have also been shown to persist in vitro in myeloid and lymphoma cell lines [57,58,59]. However, further research is required to delineate the potential of SWPV to persist in immunocompetent animals.

The in utero infection of piglets with SWPV from clinically unremarkable sows suggests that viremia occurs in immunocompetent animals, together with virus spread to reproductive tissue and vertical virus transmission through the placenta. Although the placentas from these cases were not available for further study, we did observe that the lowest ct values for SWPV apart from skin tissue were present in the umbilical cord. We were unable to consistently identify SWPV infection by qPCR in the blood of sows within the housing units in which swinepox cases occurred. Similar observations have been made previously for other poxvirus infections. Rare cases of generalized poxvirus lesions in the human fetus following in utero virus infection have been reported following both primary vaccinia virus vaccination [60,61,62] and monkeypox infections during pregnancy [63]. This is consistent with cases of intrauterine poxvirus infection in animals [64,65]. Prior to eradication, Variola virus infections resulted in the termination of 60–75% of pregnancies due to the severe consequences of systemic virus infection. Furthermore, half of the babies born alive were reported to die within two weeks, even though only 9% of newborns exhibited visible cutaneous pox lesions [66]. Further research is required to determine if SWPV is responsible for a higher case number of gestational losses and asymptomatic stillbirth in sows than previously presumed, especially given that visible poxvirus lesions are normally present on only a few of the piglets in a litter.

Estimating the real prevalence of SWPV in domestic pig populations has been challenging, as swinepox is often neglected by farmers due to the mild clinical signs in noncongenitally infected piglets. Serological testing for antibodies against SWPV is also unreliable and technically challenging, with only limited neutralization activity detected in sera from convalescent animals, which are nevertheless protected against reinfection [21,29,67]. The distribution of SWPV may be wider than assumed given the fact that SWPV infection and disruption of skin epithelium could be the underlying cause of more commonly observed secondary bacterial infections, which mask the distinctive cutaneous lesions of swinepox. Such infections can induce an exacerbation in the disease course and lead to a more severe drop in pig herd performance.

## 5. Conclusions

We have characterized two SWPV strains circulating in German wildlife and livestock animals and shown differences with respect to coding capacity within the SWPV-specific terminal region of the genome. The congenital infection of piglets and the detection of SWPV DNA in the umbilical cord suggests that the virus transmission within the domestic pig population is mediated by persistently infected asymptomatic animals. Future studies are required to determine the true prevalence and strain diversity of SWPV in domestic and wild pig species and to understand the role of comorbidities and coinfections in exacerbation of the disease course in infected animals.

## Figures and Tables

**Table 1:** List of swinepox virus infected tissue samples from domestic pigs and wild boar.

Host-ID	Species	Age of Animals	Tissue	RT-PCR (Ct Value)
101-200136	Domestic pig	1 day ^1^	FFPE skin	23.81
201-200045	Domestic pig	1 day ^1^	skin	15.78
201-200046	Domestic pig	1 day	skin	18.96
201-200047	Domestic pig	1 day	tongue	28.33
201-200047	Domestic pig	1 day	skin	19.02
201-200047	Domestic pig	1 day	lung	37.03
201-200047	Domestic pig	1 day	intestine	31.37
201-200047	Domestic pig	1 day	umbilical cord	21.43
201-200047	Domestic pig	1 day	esophagus	28.24
201-200070	Wild boar	8 weeks ^2^	skin	15.95
201-200071	Wild boar	8 weeks ^2^	skin	14.16

^1^ Euthanazied by veterinarian; ^2^ estimated age of wild boar piglets.

## Data Availability

The sequencing data presented in this study are available in GenBank, accession nos. MZ773481 (SWPV/domestic/GER/2019) and MZ773480 (SWPV/wildboar/GER/2019).

## Supplementary Materials

The following are available online at https://www.mdpi.com/article/10.3390/v13102038/s1, Figure S1: Nucleotide sequence identity observed between full genomes of different SWPV strains, Table S1: Nucleotide and amino acid changes between domestic pig and wild boar SWPV strains.
