# Supplementary material for: Swinepox Virus Strains Isolated from Domestic Pigs and Wild Boar in Germany Display Altered Coding Capacity in the Terminal Genome Region Encoding for Species-Specific Genes
Source: Viruses. 2021 Oct 9;13(10):2038. doi: 10.3390/v13102038 (PMC8538704; doi:10.3390/v13102038)
Supplement: Supplementary file 1 [file viruses-13-02038-s001.zip › viruses-1374416-supplementary.pdf]

viruses

Supplementary Materials

Swinepox Virus Strains Isolated from Domestic Pigs and Wild Boar in Germany Display Altered Coding Capacity in the Terminal Genome Region Encoding for Species-Specific Genes

SWPV strains	NC003389 SWPV/USA	MZ773481 SWPV/domestic	MZ773480 SWPV/wildboar	MW036632 SWPV/India
**NC003389 SWPV/USA**	100%	99.940%	99.922%	98.149%
**MZ773481 SWPV/domestic**	99.940%	100%	99.924%	98.146%
**MZ773480 SWPV/wildboar**	99.922%	99.924%	100%	98.140%
**MW036632 SWPV/India**	98.149%	98.146%	98.140%	100%

Figure S1. Nucleotide identity matrix comparison for swinepox virus (SWPV) strains

Table S1. Nucleotide and amino acid changes between the untranslated regions (white background) and genes (pale blue background) of different SWPV strains

Gene			nt and aa Changes Compared to SWPV /Domestic Pig/USA/2002 (NC_003389)		nt and Amino Acid Changes Compared to SWPV/Domestic pig/GER/2019 (MZ773481)
**SWPV /Domestic Pig/USA/2002 (NC_003389)**	**SWPV/ Domestic Pig/GER/2019 (MZ773481)**	**SWPV/Wild Boar/GER/ 2019 (MZ773480)**	**SWPV/Domestic Pig/GER/2019 (MZ773481)**	**SWPV/Wild Boar/GER/2019 (MZ773480)**	**SWPV/Wild Boar/GER/2019 (MZ773480)**
	U			38T>C	38T>C
				108A>G	108A>G
			163insT	163insT	
				238insT	238insT
SPV001	SPVdp001	SPVwb001		607C>T (Y10D)	607C>T (Y10D)
			710C>A		711A>C
	U		792del		793insT
SVP002	SPVdp002	SPVwb002		983A>C (Y111D)	995A>C (Y111D)
				984T>C (Y111D)	996T>C (Y111D)
	U			1327insA	1327insA
SVP004	SPVdp004	SPVwb004	2546C>T (R76K)	2549C>T (R76K)	
				2663G>A (A38V)	2663G>A (A38V)
SVP006	SPVdp006	SPVwb006	5091T>G	5094T>G	
SVP008	SPVdp008	SPVwb008	6122T>C (N255S)	6125T>C (N255S)	
				6571T>C	6571T>C
				6582G>T (Q103K)	6582G>T (Q103K)
				6588T>C (I101V)	6588T>C (I101V)
SVP009	SPVdp009	SPVwb009	6958G>T (S143Y)		6961T>G (Y143S)
			7040C>T (V116I)	7043C>T (V116I)	
	U		8151insA		8154del
SVP016	SPVdp016	SPVwb016		11878C>T (M123I)	11878C>T (M123I)
SVP017	SPVdp017	SPVwb017	12472G>A (T20I)		12474A>G (I20T)
SVP020	SPVdp020	SPVwb020-019		13061del (fusion of SPV019/SPV020)	13061del (fusion of SPV019/SPV020)
	U		13175del 5nts		13157ins 5nts
U	SPVdp020a	U	13424del (new ORF)		13426ins (truncation of ORF SPVdp021)
	U		13475del 11nts		13486ins 11nts
SPV021	SPVdp021	SPVwb021	13524T>C (K206R)		13542C>T (R206K)
SPV024	SPVdp024	SPVwb024	17243A>C (F424L)	17261A>C (F424L)	
				18392T>C	18392T>C
	U		19670del		19688insT
SPV028	SPVdp028	SPVwb028	21239T>C (E19G)		21258C>T (G19E)
	U		23082insT	23101insT	
				23110T>C	23110T>C
SPV033	SPVdp033	SPVwb033		26495G>T (Q21K)	26495G>T (Q21K)
SPV035	SPVdp035	SPVwb035		28529G>T (A53S)	28529G>T (A53S)
SPV036	SPVdp036	SPVwb036	30879C>T	30898C>T	

SPV037	SPVdp037	SPVwb037		32515del	32515del
	U			32546insA	32546insA
SPV038	SPVdp038	SPVwb038		33378C>T (A405T)	33378C>T (A405T)
SPV042	SPVdp042	SPVwb042		37366G>A (A560V)	37366G>A (A560V)
				38637T>C	38637T>C
			38632C>T (D132N)	38651C>T (D132N)	
SPV044	SPVdp044	SPVwb044	39759T>C		39778C>T
SPV046	SPVdp046	SPVwb046	42213A>G (I145V)	42232A>G (I145V)	
SPV047	SPVdp047	SPVwb047	43949T>C	43968T>C	
				44460G>A	44460G>A
SPV055	SPVdp055	SPVwb055	50246A>C (Y30S)	50265A>C (Y30S)	
SPV059	SPVdp059	SPVwb059	53332T>C	53351T>C	
SPV061	SPVdp061	SPVwb061	54988T>C (S61P)	55007T>C (S61P)	
SPV063	SPVdp063	SPVwb063	56151C>G	56020A>G	56020A>G
				56170C>G	
SPV064	SPVdp064	SPVwb064	56350G>A (V48M)		56369A>G (M48V)
			56586T>C	56605T>C	
			56667A>C	56686A>C	
SPV068	SPVdp068	SPVwb068	60143G>A (V460I)	60162G>A (V460I)	
			60433A>T	60452A>T	
			60697G>T (E644D)		60716T>G (D644E)
SPV072	SPVdp072	SPVwb072	65222G>A	65241G>A	
SPV076	SPVdp076	SPVwb076		69877T>C	69877T>C
SPV077	SPVdp077	SPVwb077	72157T>C		72176C>T
SPV079	SPVdp079	SPVwb079		73255A>G	73255A>G
SPV083	SPVdp083	SPVwb083		78742C>A (P78Q)	78742C>A (P78Q)
SPV085	SPVdp085	SPVwb085	79979A>G (M591T)	79998A>G (M591T)	
			79984T>C		80003C>T
				80255T>G	80255T>G
			80692G>T		80711T>G
SPV087	SPVdp087	SPVwb087	83187G>A	83206G>A	
			83411C>T (A302T)	83430C>T (A302T)	
			83840C>T (V159I)	83859C>T (V159I)	
SPV092	SPVdp092	SPVwb092	87971A>G (C82R)		87990G>A (R82C)
				88114T>C	88114T>C
SPV095	SPVdp095	SPVwb095	91653T>C (S123G)	91672T>C (S123G)	
SPV098	SPVdp098	SPVwb098		94691G>C	94691G>C
			95250G>T (Q226K)		95269T>G (K226Q)
			95647T>A	95666T>A	
SPV105	SPVdp105	SPVwb105	98642C>T	98661C>T	
SPV109	SPVdp109	SPVwb109	102690A>G (N173S)		102709G>A (S173N)
				102812T>C	102812T>C
				103274A>G	103274A>G
SPV111	SPVdp111	SPVwb111	103655C>A		103674A>C
SPV112	SPVdp112	SPVwb112	104067A>C (K35N)	104086A>C (K35N)	
			104130C>A		104149A>C

SPV113	SPVdp113	SPVwb113	106622T>G (Y504D)	106641T>G (Y504D)	
			106772A>C	106791A>C	
SPV115	SPVdp115	SPVwb115	109299T>G	109318T>G	
SPV116	SPVdp116	SPVwb116		110234A>G	110234A>G
	U			111576del 11nts	111576del 11nts
SPV122	SPVdp122	SPVwb122	113733G>A (A137T)	113741G>A (A137T)	
SPV123	SPVdp123	SPVwb123	114503T>C	114511T>C	
SPV126	SPVdp126	SPVwb126		117094T>A (K101I)	117094T>A (K101I)
				117103del 6nts (N106-107del)	117103del 6nts (N106-107del)
SPV131	SPVdp131	SPVwb131	120915ins 3nts (22I)	120917ins 3nts (22I)	
			121393C>T	121395C>T	
			121887A>G		121889G>A
			123410A>G		123412G>A
				123741G>A	123741G>A
			124213T>C	124215T>C	
			124544G>A (V1231I)	124546G>A (V1231I)	
			124979G>A (V1376I)	124981G>A (V1376I)	
				125128G>A (D1425N)	125128G>A (D1425N)
				126256C>A (H1801N)	126256C>A (H1801N)
SPV132	SPVdp132	SPVwb132	127438A>C (H242P)	127440A>C (H242P)	
			127656T>C (Y315H)	127658T>C (Y315H)	
SPV134	SPVdp134	SPVwb134		128978C>A	128978C>A
SPV136	SPVdp136	SPVwb136		130126T>C	130126T>C
SPV139	SPVdp139	SPVwb139		133889C>A	133889C>A
SPV141	SPVdp141	SPVwb141	136344C>A		136346A>C
	U		139973insT	137036del; 137079C>T; 138626del; 139973insT	137036del; 137079C>T; 138626del
SPV144	SPVdp144	SPVwb144		140046T>C (V19A)	140046T>C (V19A)
	U		142452insA		142454del
SPV146	SPVdp146	SPVwb146	142606C>T		142605T>C
SPV147	SPVdp147	SPVwb147		143783C>T (A38V)	143783C>T (A38V)
			143898G>A (R76K)	143897G>A (R76K)	
U		SPVwb149		145110insT (+8 amino acids at gene start)	145110insT (+8 amino acids at gene start)
SPV149	SPVdp149	SPVwb149		145450A>G (Y111D)	145450A>G (Y111D)

				145451T>G (Y111D)	145451T>G (Y111D)
	U		145643del		145644insA
SPV150	SPVdp150	SPVwb150	145734G>T (D10Y)		145735T>G (Y10D)
				145839G>A	145839G>A
	U			146201insA	146201insA
			146275insA	146277insA	
				146338T>C	146338T>C
				146408A>G	146408A>G
